# Book Review

**Published:** 1937

**Authors:** 


### Reviews of Books

**What is Osteopathy?** By C. Hill, M.D., D.P.H., and H. A. Clegg, M.B., M.R.C.P. Pp. xix., 217. Illustrated. London: J. M. Dent & Sons Ltd. 1937. Price 7s. 6d.—As the title suggests, this book is written for the non-medical public, and its purpose is to examine the theory and practice of osteopathy, showing that if this cult be right then medicine as generally understood is wrong. Mr. H. G. Wells, strangely called in to write a preface, and seemingly on the strength of having been at once cured of a lameness, "after months of fumbling on the part of normal medical men," by Sir Herbert Barker, says that "the book tells the story of osteopathy calmly, but with a twinkle of amusement that sometimes gives place to a twinkle of indignation. Plainly osteopathy, considered as a system of medicine or as anything more than the manipulative side of surgery, is impudent balderdash." And Dr. Morris Fishbein has put the matter in a nutshell in his terse statement that "osteopathy of to-day is essentially an attempt to enter the practice of medicine by the back door." An account of the life and practice of the American Andrew T. Still, the "father of osteopathy," is given first. Then follow copious verbatim extracts from the investigation conducted by the select committee of the House of Lords in 1935, when the osteopaths sought State recognition and registration. Osteopathy was defined by the promoters of the Bill as "that system of the healing art which places the chief emphasis on the integrity of the body mechanism as being the most important single factor to maintain the well-being of the organism in health and disease." This claim is analysed and illustrated. But "the committee found on the evidence before them that the claim of the osteopaths to treat all diseases as set out in the Bill had not been established, and that it would not be proper for Parliament to recognize osteopathic practitioners as qualified on a similar footing to that of registered medical practitioners, to diagnose and treat all human complaints." The committee recommended that the Bill should not be further proceeded with. It may be pointed out that manipulation of the spinal column, that is chiropraxy, appears to play a large part in osteopathic treatments, which probably accounts for the confusion of the two cults in the lay mind. The chiropractors (estimated to number 16,000 in the United States, 1932) ascribe all diseases, from tabes or typhoid to diabetes or diphtheria, to "subluxations" of the vertebræ alone. The osteopaths (7,650 in number) consider other joints as well. All these practitioners, together with Christian Science Healers (10,000), are "state registered" in the U.S.A. The authors of this book emphasize that they do not deny that osteopathic manipulation may do good in certain conditions, but conditions which respond to treatment at the skilled hands of the manipulative surgeon and the bone-setter; and if the latter would only restrict his therapeutic claims to such special conditions, without erecting a new theory of health and disease on unproven hypotheses, the medical profession would have no excuse for not listening to him. No more convincing indictment of a charlatanism has been framed. Finally, the authors briefly indicate how to qualify to become a recognized specialist in bone-setting, should a narrow path be preferred in our craft.

**Skin Diseases in General Practice.** Third Edition. By H. HALDIN-DAVIS, D.M., F.R.C.P. Pp. xiii., 400. Illustrated. London: Oxford University Press (Humphrey Milford). 1937. Price 17s. 6d.—The third edition of this text-book maintains its reputation as a sound practical work on the diagnosis and treatment of the common diseases of the skin. The arrangement on a basis of distribution rather than according to ætiology is probably appreciated by the practitioner who wishes to look up rapidly the differential diagnosis of an eruption which he is called upon to treat, while a useful chapter on "Modern Methods of Treatment" will also be found of interest. One is rather surprised to find no mention of the pioneer work of Whitfield in the discussion of the phenomenon of auto-sensitization in eczema, and the author's opinion that wet dressings and lotions are not indicated in acute eczematous eruptions is at variance with the practice of most dermatologists. There are seven coloured plates and ninety black and white illustrations. This edition, which is well written and well produced, should be as popular as its predecessors.

**Diseases of the Eye.** By E. WOLFF, M.B., B.S., F.R.C.S. Pp. xi., 234. Illustrated. London : Cassell & Co. Ltd. 1937. Price 15s.—Intended for the use of students and practitioners, this new guide to ophthalmology should make a special appeal to the beginner in the subject. With the aid of annotated anatomical drawings and a large number of other illustrations, the author has succeeded in presenting his subject in a way which should simplify considerably the many problems met with by the inexperienced. The illustrations are, in fact, an outstanding feature of the book, of high quality and excellently reproduced. The large pages (of quarto size) enable them to be shown to advantage and to be placed conveniently in relation to the text. In addition to chapters on the diseases affecting the various structures of the eye, the volume contains sections dealing with ophthalmoscopy, the field of vision, errors of refraction, congenital anomalies, injuries, the eye complications of certain general diseases and a representative selection of the more usual operations. The descriptions of clinical methods and of the subject in its various aspects are clearly written, the teaching is up to date, and the lines of treatment laid down are in most instances modern and orthodox. Although the amount of space devoted to descriptions of unusual conditions is rather more than one would expect in an elementary text-book, adequate attention is paid to the commoner and more important ailments. This is a distinctly attractive text-book, and can be cordially recommended to those for whom it has been written.

**Material Medica for Nurses.** Fourth Edition. By A. M. CRAWFORD, M.D., F.R.F.P.S.G. Pp. viii., 105. London: H. K. Lewis & Co. 1937. Price 3s. 6d.—This handy little guide for nurses has undergone few changes in the fourth edition. A few paragraphs on local and general anæsthetics have been added. There is a new chapter on vitamins, in which the complexity of B is alluded to, but no reference is made to any variations in the constitution of D. However, the information is probably sufficient for nurses. Another new chapter deals with the sugars. This seems to give less information than nurses might need. The rapid conversion of sucrose into dextrose in the body is not mentioned. The description of the conditions for which glucose is commonly administered omits all mention of surgical and operative emergencies. No reference is made to the use of sugars in artificial feeding of infants, a matter of great interest to nurses—in fact, *lactose* is dismissed as being "used to give bulk to other powders of minute dosage." *Hiatus valde deflendus!*

**A Criticism of Nursing Education with Suggestions for Constructive Reform.** By H. BALME, M.B., F.R.C.S., D.P.H. Pp. viii., 73. London: Oxford University Press (Humphrey Milford). 1937. Price 2s. 6d.—This book certainly lets a breath of fresh air into the subject of the education of the nurse in hospital. Every suggestion made is practical and possible, and would if carried out honestly enlist better educated girls or women for the service. The heavy work which a probationer undergoes for the first year of her hospital life at present would not be hard if given to attendants who are used to such work. The idea of a probationer being a student for one year in a nursing college—paying fees for instruction and being carefully taught—would certainly be an innovation which would surprise the kind of sister so well described in Chapter IV, page 22. The Ministry of Health is an open-minded body; it would be necessary to enlist its help and *Authority* before such a scheme as that outlined in Chapter VII could be carried out. Anyone interested in increasing the number of applicants for hospital training of a more educated class would do well to read and digest the ideas conveyed by the writer. The unnatural discipline maintained in the present-day schools of nursing is unnecessary; if made lighter it would undoubtedly make the high-spirited woman of to-day more ready to devote herself to the truly interesting and absorbing career of a trained hospital nurse.

